# 柱前衍生-超高效液相色谱法测定3种奶粉中18种氨基酸

**DOI:** 10.3724/SP.J.1123.2020.07008

**Published:** 2021-05-08

**Authors:** Li QU, Shuqing GU, Jiaqi ZHANG, Chaomin ZHAO, Xiaojun DENG

**Affiliations:** 1.上海海关动植物与食品检验检疫技术中心, 上海 200135; 1. Technical Center for Animal Plant and Food Inspection and Quarantine, Shanghai Customs, Shanghai 200135, China; 2.复旦大学, 上海 200032; 2. Fudan University, Shanghai 200032, China

**Keywords:** 超高效液相色谱, 柱前衍生, 6-氨基喹啉-*N*-羟基琥珀酰亚胺氨基甲酸酯, 氨基酸, 牛奶粉, 羊奶粉, 骆驼奶粉, ultra performance liquid chromatography (UPLC), pre-column derivatization, 6-aminoquinoline-*n*-hydroxysuccinimide carbamate (AQC), amino acids, milk powder, goat milk powder, camel milk powder

## Abstract

近年来羊奶粉和骆驼奶粉备受消费者青睐,它们具有潜在的低致敏性,因此成为牛乳不耐受人群尤其是婴幼儿的母乳替代品,其营养价值备受关注。牛奶粉、羊奶粉和骆驼奶粉中氨基酸含量的比较研究鲜有报道。利用酸水解得到游离氨基酸,选择6-氨基喹啉-*N*-羟基琥珀酰亚胺氨基甲酸酯(AQC)进行柱前衍生,超高效液相色谱分离并检测,外标法定量。18种氨基酸在各自线性范围内线性关系良好,相关系数(*r* ^2^)大于0.999;以3倍和10倍信噪比(*S/N*)确定方法的检出限(LOD)和定量限(LOQ),分别为1.3~2.5 (mg/100 g)和3.9~7.5 (mg/100 g)。方法验证采用奶粉标准参考物质SRM 1849a,测定值符合其含量范围,6次测定值的相对标准偏差(RSD)为2.04%~3.65%。采用建立的方法分别对市售和网购的牛奶粉、羊奶粉和骆驼奶粉进行18种氨基酸成分和含量分析,旨在从氨基酸角度对这3种不同来源乳品进行对比。该方法快速,灵敏度高,准确可靠,适用于不同基质乳粉中18种氨基酸成分和含量的确定。

## Abstract (en)

In recent years, goat milk powder and camel milk powder have gained popularity among consumers. Due to their potential low allergenicity, these milk powders have become a substitute for breast milk, especially for infants, and for people with lactose intolerance. In this paper, a method was developed for the simultaneous determination of 18 amino acids (AAs), histidine (His), serine (Ser), arginine (Arg), glycine (Gly), aspartic acid (Asp) combined with asparagine (Asn), glutamic (Glu), glutamine (Gln), threonine (Thr), alanine (Ala), proline (Pro), lysine (Lys), tyrosine (Tyr), methionine (Met), valine (Val), isoleucine (Iso), leucine (Leu), and dimer of cysteine (Cys) combined with cysteine (L-Cys-Cys), phenylalanine (Phe), taurine (Tau) in milk, goat milk, and camel milk power. The aim of the research was to compare the three kinds of milk powder from the perspective of the constituent amino acids. Therefore, the amino acid compositions and contents were compared. Thus, 2.0 g of the sample was accurately weighed, added to 16 mL H_2_O, and mixed thoroughly. Then, 200 mg of the sample was weighed in a glass tube with a stream of nitrogen to displace oxygen. The samples were hydrolyzed in HCl for 24 h at 110 ℃. Then, the amino acids were pre-column derivatized by 6-aminoquinoline-*n*-hydroxysuccinimide carbamate (AQC). In precolumn derivatization combined with reverse-phase chromatography, both 2,4-dinitrofluorobenzene (DNFB) and phenylisothiocyanate (PITC) can react with primary amines and secondary amines. However, the derivatization time is approximately 1 h. In contrast, the derivatization time of AQC was greatly shortened. Derivatization led to the conversion of free amino acids into highly stable derivatives, which were separated by ultra performance liquid chromatography (UPLC) with UV detection at 260 nm and quantified by the external standard method. The samples were separated on a BEH C_18_ column (150 mm×2.1 mm, 1.7 μm) at a flow rate of 0.4 mL/min. The calibration curves showed good linearity, with correlation coefficients greater than 0.999. The limits of detection (LODs) and limits of quantification (LOQs) of the 18 amino acids were 1.3-2.5 (mg/100 g) and 3.9-7.5 (mg/100 g), respectively. Quality control samples of SRM 1849a were used as the reference material. The results were in accordance with the content range. The RSDs ranged from 2.04% to 3.65%. Furthermore, the developed method was successfully applied to determine the types and concentrations of amino acids in 11 samples purchased from local markets in Shanghai and online shops. Abundant amino acids were detected in the three types of milk powder. While all the milk powder samples contained 18 types of amino acids, Tau was not detected in some of the goat and camel milk powder samples. Total essential amino acids (TEAA) in total amino acids (TAA) of milk powder was the highest of all. The TEAA values of TAA in the goat and camel milk powders were similar. The developed method requires only 22 min for the separation of 18 amino acids. This method is suitable for the large-scale analysis of milk powder samples, and it demonstrates high sensitivity and accuracy for the determination and confirmation of the 18 amino acids in different types of milk powders.

氨基酸(amino acids, AA)是大分子蛋白质的基本组成单位,是生物代谢过程中的重要物质,氨基酸的种类及含量影响蛋白质的形态、特性和营养价值。牛乳营养丰富,是婴幼儿阶段重要的营养来源,但是其中含有20多种过敏蛋白质,其中酪蛋白和乳清蛋白可引起大多数过敏反应^[1]^。羊奶和羊奶制品似乎具有潜在的低致敏性,因此成为牛乳不耐受人群,尤其是婴幼儿的母乳替代品^[2,3]^。骆驼奶的蛋白质组成和结构与牛乳不同,具有不同的功能和药用特性^[4]^,骆驼奶与母乳相似,骆驼奶具有较高的消化率和较低的婴儿过敏发生率^[5]^。Navarrete-Rodríguez等^[6]^证实骆驼奶对一岁以上患有牛乳蛋白过敏症(CMPA)的患者是安全和可耐受的,被认为是一种很好的母乳替代品。由于上述这些优势,近年来羊奶粉和骆驼奶粉备受消费者青睐,其价格也比牛奶粉高。为了探究3种不同乳品的差异,本研究从氨基酸角度进行氨基酸成分分析和含量比较,为研究3种来源乳品营养价值差异奠定了基础。

目前氨基酸检测的方法有分光光度法^[7]^、氨基酸分析仪法^[8]^、高效液相色谱法^[9]^、液相色谱-串联质谱法(LC-MS/MS)^[10,11]^等,其中我国国家标准GB/T 5009.124-2016《食品中氨基酸的测定》以茚三酮为柱后衍生剂,采用氨基酸分析仪测定。该方法所需衍生剂、色谱柱及配套流动相的采购成本较高,且该仪器普及性低,检测时间较长。LC-MS/MS可以解决多种氨基酸分离度差以及引入衍生试剂后分离难度大等问题^[12]^,常用于血液^[13]^、尿液^[14]^、烟草^[15,16]^等样品基质的分析。针对乳制品,高效液相色谱法比较普遍,由于选取的衍生试剂不同,大多衍生时间和分析时间较长,本研究利用6-氨基喹啉-*N*-羟基琥珀酰亚胺氨基甲酸酯(AQC)作为衍生化试剂,大大缩短前处理时间,在22 min内可以分离18种氨基酸。方法灵敏度高,重复性好,结果可靠,可用于不同基底乳粉中18种氨基酸的定量检测。

## 1 实验部分

### 1.1 仪器、试剂与材料

Waters ACQUITY UPLC型超高效液相色谱仪,配有PDA检测器和Empower色谱数据工作站(美国Waters公司); ME104T电子分析天平(精度为0.1 mg,美国Mettler Toledo公司); Vortex-Genie2涡旋振荡仪(美国Scientific Industries公司); DKN 812C烘箱(日本Yamato公司)。

组氨酸(His)、丝氨酸(Ser)、精氨酸(Arg)、甘氨酸(Gly)、天冬氨酸(Asp)、谷氨酸(Glu)、苏氨酸(Thr)、丙氨酸(Ala)、脯氨酸(Pro)、赖氨酸(Lys)、酪氨酸(Tyr)、甲硫氨酸(Met)、缬氨酸(Val)、异亮氨酸(Iso)、亮氨酸(Leu)、半胱氨酸(Cys)、苯丙氨酸(Phe)、L-胱氨酸(L-Cys-Cys)、牛磺酸(Tau)、苯酚、3,3'-二硫代二丙酸、甲酸铵均购自美国Sigma公司;氢氧化钠、盐酸、硼酸均购自国药集团化学试剂有限公司;AQC(Cas号:148757-94-2)购自美国TRC公司;Waters AccQ·Tag^TM^流动相购自美国Waters公司;0.22 μm滤膜购自上海安谱科技股份有限公司;实验室用水均由Milli-Q超纯水系统(美国Millipore公司)制得。

牛奶粉和羊奶粉均购自上海市场,骆驼奶粉购于网络;标准参考物质SRM 1849a奶粉样品购自美国国家标准与技术研究院(NIST)。

### 1.2 样品前处理

1.2.1 水解

采用酸水解法,准确称取2.0 g样品,加入16 g水,充分溶解后,准确称取200 mg样液,置于10 mL带螺纹玻璃瓶中,依次加入800 μL水、600 μL 1% 3,3'-二硫代二丙酸、600 μL 0.2 mol/L盐酸、2.8 mL 0.1%苯酚盐酸,混匀后持续充入氮气1 min,置于110 ℃烘箱中24 h,于第2天取出并冷却至室温,待带螺纹玻璃瓶的残渣沉到瓶底,吸取0.2 mL澄清样液至1.5 mL离心管中,依次加入0.2 mL 6 mol/L NaOH和0.4 mL 0.1 mol/L盐酸,混合均匀,过水相滤膜后待衍生。

1.2.2 衍生

衍生试剂的配制:称取1.0 mg AQC粉末,置于1.5 mL离心管中,而后加入1 mL纯乙腈,涡旋混匀,于55 ℃加热,使其全部溶解,在变色硅胶容器中密封储存一周。

吸取70 μL 0.4 mol/L硼酸缓冲液(pH 8.8),置于1.5 mL离心管中,吸取10 μL 1.2.1节制备的待衍生的样液,混合均匀后,边涡旋边加入20 μL配制好的AQC衍生试剂,充分混合后,于55 ℃加热10 min,将衍生好的试样装入内插管中,超高效液相色谱仪检测,外标法定量。

### 1.3 色谱条件

色谱柱:BEH C_18_柱(150 mm×2.1 mm, 1.7 μm);柱温:50 ℃;流速:0.4 mL/min;流动相A: AccQ·Tag^TM^流动相;流动相B: 0.2‰甲酸乙腈溶液。梯度洗脱程序:0~5.5 min, 0.1%B; 5.5~15.2 min, 0.1%B~9.1%B; 15.2~20.5 min, 9.1%B~21.2%B; 20.5~21.26 min, 21.2%B~59.6%B; 21.26~21.29 min, 59.6%B~90%B; 21.29~22.84 min, 90%B; 22.84~26.00 min, 90%B~0.1%B; 26.00~32.00 min, 0.1%B。进样量:1 μL;检测波长:260 nm。

## 2 结果与讨论

### 2.1 色谱方法优化

本方法考察了BEH C_18_柱(150 mm×2.1 mm, 1.7 μm)和T_3_柱(150 mm×3.0 mm, 1.8 μm)。结果表明,虽然两者都是耐高水相色谱柱,但是采用T_3_柱分离后,目标物色谱峰峰形较差,响应较低,而BEH C_18_柱柱效高,目标物色谱峰峰形窄且尖锐,能够达到较好的分离度,可以实现22 min内18种氨基酸的基线分离(见图1a)。在酸水解过程中,氨基酸经过AQC衍生后,谷氨酰胺(Gln)和天冬酰胺(Asn)分别转化为Glu和Asp。因此,Glu值表示Glu和Gln的组合值;Asp值表示Asp和Asn的组合值。Barkholt等^[17]^研究表明在水解过程中加入3,3'-二硫代二丙酸可以将Cys和L-Cys-Cys转化为*S*-2-羧乙基硫半胱氨酸(xCys),所得到的xCys可与其他氨基酸分离,从而进行定量。如图1b所示,xCys值表示Cys和L-Cys-Cys的组合值。

**图 1:**
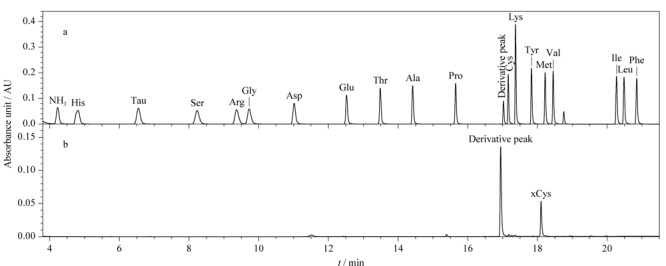
(a)18种氨基酸和(b)xCys的色谱图

### 2.2 衍生试剂的选择

蛋白质在盐酸环境下110 ℃水解24 h,其中添加0.1%苯酚的目的是防止酪氨酸卤化;55 ℃烘箱中加热10 min,目的是将酪氨酸衍生时的副产物快速转化为单一产物。氨基酸不具有紫外吸收性质,需要经过衍生化过程才能被仪器捕捉到,异硫氰酸苯酯(PITC)和2,4-二硝基氟苯(DNFB)均可与一级胺、二级胺反应,是氨基酸常用的柱前衍生剂^[18,19]^,使用这两种试剂衍生的时间大约需要1 h左右,而使用AQC衍生仅需要1 s,这大大缩短了衍生化时间。也正是因为AQC的衍生化时间非常短,所以样品中加入衍生化试剂时,需要一边低速涡旋一边加入,使衍生化试剂与样品接触面积最大,确保样品中氨基酸全部衍生。否则会导致样品还未完全与衍生试剂反应,衍生试剂就水解了。AQC极容易水解所以配制好后应当密封保存在干燥容器内。

### 2.3 方法学验证

2.3.1 标准曲线、线性范围和检出限

除Cys外,配制其他17种氨基酸标准溶液,浓度分别为0、0.5、1.0、5.0、10、20 nmol/mL;同时配制0、0.5、1.0、2.5、5.0、10 nmol/mL Cys标准溶液,按上述色谱条件进行分析。

以18种氨基酸换算后的质量浓度为横坐标(*X*, μg/L)、峰面积为纵坐标(*Y*),绘制标准曲线。以3倍和10倍信噪比(*S/N*)确定方法的检出限(LOD)和定量限(LOQ),各组分的线性方程、相关系数(*r*^2^)见表1。结果表明,各组分的线性关系良好,可满足实际检测的需要。

**表 1:** 18种氨基酸的相对分子质量、线性方程、相关系数、检出限和定量限

Compound	*M*_r_	Linear range/(nmol/mL)	Linear equation	*r* ^2^	LOD/(mg/100 g)	LOQ/(mg/100 g)
His	155.16	0-20	*Y*=7.65×10^3^*X*-5.77×10^2^	0.9998	2.5	7.5
Tau	125.15	0-20	*Y*=8.31×10^3^*X*-1.78×10^2^	0.9999	2.4	7.2
Ser	105.09	0-20	*Y*=7.64×10^3^*X*+8.15×10^2^	0.9998	2.2	6.6
Arg	174.20	0-20	*Y*=7.56×10^3^*X*+1.17×10^3^	0.9997	2.5	7.5
Gly	75.07	0-20	*Y*=7.54×10^3^*X*+4.50×10^2^	0.9998	2.1	6.3
Asp	133.11	0-20	*Y*=7.68×10^3^*X*+5.14×10^2^	0.9998	2.2	6.6
Glu	147.13	0-20	*Y*=7.22×10^3^*X*+2.28×10^3^	0.9997	1.6	5.0
Thr	119.12	0-20	*Y*=7.90×10^3^*X*+8.52×10^2^	0.9998	1.4	4.5
Ala	89.10	0-20	*Y*=7.84×10^3^*X*+1.61×10^3^	0.9998	1.3	3.9
Pro	115.13	0-20	*Y*=7.34×10^3^*X*+2.10×10^3^	0.9997	2.1	6.3
Lys	146.19	0-20	*Y*=1.34×10^4^*X*+1.66×10^2^	0.9997	1.3	3.9
Tyr	181.19	0-20	*Y*=8.20×10^3^*X*+2.90×10^3^	0.9996	2.1	6.3
Met	149.21	0-20	*Y*=8.07×10^3^*X*+1.91×10^3^	0.9998	1.7	5.1
Val	117.15	0-20	*Y*=8.21×10^3^*X*-1.34×10^2^	0.9996	1.3	4.0
Iso	131.18	0-20	*Y*=8.13×10^3^*X*+1.62×10^3^	0.9997	2.2	4.5
Leu	131.18	0-20	*Y*=8.23×10^3^*X*+2.05×10^3^	0.9997	2.3	6.9
Phe	165.19	0-20	*Y*=8.09×10^3^*X*+1.12×10^3^	0.9998	2.1	4.2
xCys	240.30	0-10	*Y*=1.31×10^4^*X*-2.09×10^2^	0.9999	2.5	7.5

*Y*: peak area; *X*: mass concentration, μg/L.

2.3.2 方法可行性验证

为了验证方法的可行性,以奶粉标准参考物质1849a为对象进行测定,平行测定6次。实验表明,测定结果符合证书中范围值,6次平行测定值的RSD为2.04%~3.65%,说明该方法准确可靠。

### 2.4 实际样品检测

采用建立的方法分别对上海市售及电商销售的11个批次的牛奶粉、羊奶粉和骆驼奶粉进行18种氨基酸分析,并对其总氨基酸(TAA)、必需氨基酸(EAA)和总必需氨基酸(TEAA)进行统计。结果表明,牛奶粉、羊奶粉和骆驼奶粉中均检测到丰富的氨基酸,种类齐全,基本覆盖18种氨基酸,但是个别羊奶粉和骆驼奶粉未检出牛磺酸。

2.4.1 同一品牌不同阶段牛奶粉氨基酸组成

此次测试的上海市售的同一品牌不同阶段牛奶粉中均检测到了18种氨基酸,各氨基酸含量占总氨基酸含量的百分比(以下简称含量百分比)见表2。His、Ser、Arg、Glu、Pro、Tyr、Phe随段数增加含量百分比逐渐升高;Tau、Gly、Asp、Ala、Leu和xCys随段数升高,含量百分比逐渐下降;Thr、Lys、Met、Val、Iso的含量百分比趋势和段数并无关联。在3个阶段牛奶粉中,Tau含量百分比差别最小,Glu差别最大。牛奶粉一段至三段的TEAA占TAA的比例分别为40.2%、39.4%和38.2%,均大于联合国粮食组织(FAO)和联合国世界卫生组织(WHO)推荐标准(36.0%),属于优质蛋白质资源。

**表 2:** 同一品牌不同阶段牛奶粉、同品牌同阶段牛奶粉和羊奶粉、3种不同品牌羊奶粉、 不同产地骆驼奶粉中18种氨基酸占总氨基酸的百分含量

Compound	Milk powder of the same brand at different stages				Different powder of the same brand at stage 1			Different brands of milk powder of goat				Different origins of milk powder of camel			
	Stage 1	Stage 2	Stage 3		Milk powder	Goat powder		Brand 1	Brand 2	Brand 3		Xinjiang 1		Xinjiang 2	Dubai
His^*^	1.97	2.14	2.25		1.94	2.40		2.49	2.44	2.49		2.25		2.28	2.49
Tau	0.39	0.28	0.25		0.31	0.17		0.17	0.21	/		0.21		0.21	/
Ser	4.74	5.11	5.23		5.11	5.22		5.10	5.04	5.06		5.21		5.25	5.03
Arg	2.74	2.90	2.98		2.35	2.64		2.45	2.47	2.84		3.03		2.95	3.89
Gly	2.43	1.89	1.87		1.68	1.64		1.63	1.69	1.75		1.87		1.80	1.44
Asp	9.11	8.72	7.94		9.13	7.92		7.90	7.65	7.33		8.34		8.31	7.08
Glu	19.0	20.1	21.0		20.2	21.2		20.9	20.9	22.0		20.7		20.7	21.8
Thr^*^	5.10	5.26	5.03		5.46	5.43		5.37	5.25	4.64		4.90		4.92	4.80
Ala	4.42	4.02	3.88		4.19	3.75		3.79	3.62	3.13		3.76		3.72	2.50
Pro	6.93	8.03	8.52		7.70	9.98		9.85	9.89	10.1		8.40		8.59	10.3
Lys^*^	8.93	8.54	8.62		8.30	7.60		8.15	8.12	7.92		8.23		8.15	7.41
Tyr	3.53	3.93	4.52		3.52	3.11		3.83	4.00	4.23		4.43		4.32	4.57
Met^*^	2.25	2.34	1.27		2.26	2.16		2.28	2.45	2.50		2.49		2.51	2.84
Val^*^	6.01	5.94	6.06		6.14	6.79		6.67	6.87	6.56		6.03		6.12	6.21
Iso^*^	5.37	5.49	5.38		5.72	5.05		4.92	4.84	4.65		5.25		5.26	5.22
Leu^*^	10.6	9.66	9.58		9.98	9.18		8.97	9.02	9.20		9.41		9.44	9.21
Phe^*^	3.84	4.17	4.33		4.11	4.57		4.40	4.42	4.73		4.28		4.21	4.38
xCys	2.67	1.54	1.29		1.86	1.25		1.15	1.13	0.90		1.23		1.29	0.78
TAA/(mg/100 g)	11731	17116.5	19584		12777	15698		16029	16452	28806		20531		20108	24073
TEAA/(mg/100 g)	4716	6738	7480		5087	6060		6225	6415	10937		7917		7777	9190
TEAA in TAA/%	40.2	39.4	38.2		39.8	38.6		38.8	39.0	38.0		38.6		38.7	38.2

* essential amino acid; TEAA: total essential amino acids;/: no detected.

2.4.2 同一品牌1段牛奶粉和羊奶粉氨基酸组成

在同一品牌一段牛奶粉和羊奶粉中均检测到了18种氨基酸,羊奶粉中的必需氨基酸里只有His、Val和Phe的含量百分比高于牛奶粉。在非必需氨基酸中,羊奶粉中Tau、Asp、Ala、Tyr、Lys和xCys的含量百分比均低于牛奶粉,只有Glu和Pro高出牛奶粉。

一段婴儿奶粉是婴儿营养的重要来源,牛奶粉和羊奶粉中TEAA占TAA的比例分别为39.8%和38.6%,属于优质蛋白质资源,但未见羊奶粉氨基酸成分含量的明显优势。

2.4.3 3种不同品牌羊奶粉氨基酸组成

分析了3种不同品牌的羊奶粉,TEAA占TAA的比例为:品牌一38.8%、品牌二39.0%、品牌三38.0%,说明3种羊奶粉属于优质蛋白质资源。有两个品牌均检测到18种氨基酸;另一品牌没有检测到Tau,但其Glu的含量百分比较其他两品牌高2.0%。

2.4.4 骆驼奶粉氨基酸组成

此次分析的3种骆驼奶粉中,新疆产两款驼奶粉的18种氨基酸含量百分比比较接近,可能与骆驼品种、生长环境和饲料有关。迪拜产骆驼奶中必需氨基酸Lys含量百分比较新疆驼奶低1%,其他必需氨基酸较新疆驼奶没有太大差异。迪拜产骆驼奶中非必需氨基酸Arg、Glu和Pro含量百分比均高于新疆驼奶粉1%~2%,而Ser、Gly、Asp、Ala、xCys含量百分比均低于新疆驼奶粉0.2%~1%。新疆1号、新疆2号和迪拜驼奶粉中TEAA占TAA的比例较为相近,分别为38.6%、38.7%和38.2%,属于优质蛋白质资源。但是进口的迪拜产骆驼奶粉中,没有检出Tau。

2.4.5 3种基质奶粉中氨基酸组成

根据上述实验数据,选择牛奶粉、羊奶粉和骆驼奶粉进行氨基酸含量比对。结果表明,牛奶粉中TEAA占TAA百分比较骆驼奶粉和羊奶粉要稍高,骆驼奶粉和羊奶粉的比较接近,当然奶粉种类众多,未能全部抽检,该结果仅针对受试样品。部分品牌骆驼奶粉和部分品牌羊奶粉没有检出Tau,它是母乳中含量丰富的游离氨基酸,被认为是具有神经保护作用,能够促进宝宝的大脑发育^[20]^,提高宝宝自身抵抗力,同时还能预防疾病,缺乏牛磺酸则会造成多种危害,比如智力发育不全,可能引起神经发育障碍疾病,例如天使综合征^[21]^。胎儿需要从母体获取所需的牛磺酸,如果母乳不足,需要依靠配方奶粉维持营养,选择羊奶粉和骆驼奶粉时,应当看清营养标签中是否强化了牛磺酸,以保证营养均衡。

## 3 结论

本研究利用基于AQC衍生的超高效液相色谱法在22 min内可同时测定18种氨基酸成分。与传统方法相比,该方法采用AQC衍生,衍生时间大大缩短,前处理步骤简便。方法学考察及利用该方法对上海市售和网络销售的牛奶粉、羊奶粉和骆驼奶粉进行氨基酸成分分析后表明,该方法具有实用性和特异性强、结果稳定可靠等优点,能够满足乳粉中氨基酸成分分析需求,为我国口岸进口的进境奶制品进行监测,检验检疫行业的监控、筛查和确证、风险评估等研究提供了技术支持。
